# Double-Focusing Thermal Triple-Axis Spectrometer at the NCNR

**DOI:** 10.6028/jres.117.002

**Published:** 2012-02-02

**Authors:** J. W. Lynn, Y. Chen, S. Chang, Y. Zhao, S. Chi, W. Ratcliff, B. G. Ueland, R. W. Erwin

**Affiliations:** 1 NIST Center for Neutron Research, National Institute of Standards and Technology, Gaithersburg, MD 20899-6102; 2 Department of Materials Science and Engineering, University of Maryland, College Park, MD 20742

**Keywords:** Inelastic neutron scattering, neutron diffraction, polarized neutrons, position sensitive detector, thermal neutron triple-axis spectrometer

## Abstract

The new thermal triple-axis spectrometer at the NIST Center for Neutron Research (NCNR) is located at the BT-7 beam port. The 165 mm diameter reactor beam is equipped with a selection of Söller collimators, beam-limiters, and a pyrolytic graphite (PG) filter to tailor the beam for the dual 20×20 cm^2^ double-focusing monochromator system that provides monochromatic fluxes exceeding 10^8^ n/cm^2^/s onto the sample. The two monochromators installed are PG(002) and Cu(220), which provide incident energies from 5 meV to above 500 meV. The computer controlled analyzer system offers six standard modes of operation, including a diffraction detector, a position-sensitive detector (PSD) in diffraction mode, horizontal energy focusing analyzer with detector, a **Q**-E mode employing a flat analyzer and PSD, a constant-E mode with the analyzer crystal system and PSD, and a conventional mode with a selection of Söller collimators and detector. Additional configurations for specific measurement needs are also available. This paper discusses the capabilities and performance for this new state-of-the-art neutron spectrometer.

## 1. Introduction

The NCNR has operated as many as four thermal triple-axis instruments over the years, that typically were developed in the early stages of the facility and with quite limited budgets. As the NCNR developed into a national user facility a modernization of the thermal neutron spectrometers became essential. As part of this modernization, a new state-of-the-art triple-axis instrument has been designed and is now installed at the BT-7 thermal beam port. In addition, a second spectrometer of similar design is under development to be installed at another thermal beam port. These new instruments will take full advantage of the large 165 mm diameter beam tubes, with two interchangeable 20×20 cm^2^ double focusing monochromators that provide 400 cm^2^ in reflecting area for each monochromator. A pyrolytic graphite (PG) monochromator will be available for both instruments, and for BT-7 the second monochromator is Cu(220). Ge(311) will also be available for at least one of the instruments in the future. The analyzer system uses PG, with horizontal focusing capabilities in a variety of configurations, and together these new capabilities can provide signals that are two orders-of-magnitude larger than available with the original thermal triple-axis instruments.

## 2. Overview of the Design

**Fig. 1:**
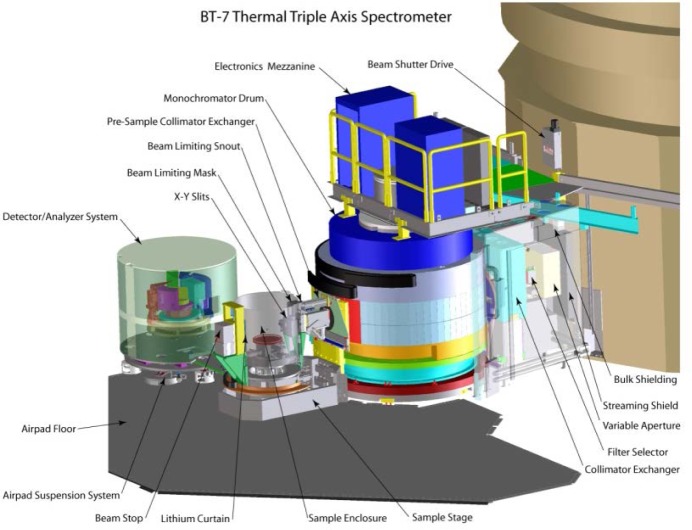
Cut-away layout of the new instrument. The size of the beam exiting from the source can be varied by a set of *x-y* slits, and can pass through a filter and a system with a selection of horizontal collimations, before striking the double-focusing monochromator. The monochromatic beam impinges on the sample table that is cantilevered from the monochromator drum. The analyzer system is on air pads.

A schematic of the spectrometer is shown in Fig. 1. On the new thermal instruments, the experimental beam shutter is contained within the biological shield of the source, and simply has two positions, open and closed. The beam shutter opening is 6.4 cm wide at the exit, opening to 9 cm on the end toward the source to fully illuminate the Söller collimators in the reactor beam, as well as to allow for horizontal focusing of the monochromator. The vertical opening is 16 cm. The shutter operates between the two positions in about eleven seconds.

The next element is the variable apertures to reduce the size of the beam when needed. The four blades, two horizontal and two vertical, are each composed of compressed ^6^LiF on the surface toward the source, with a 10 cm thick aluminum frame holder behind this filled with B_4_C. Each pair of blades of the aperture open symmetrically about the beam center to restrict the beam size horizontally and vertically. The next element is the filter, which is a tunable pyrolytic graphite (PG) filter system. No cryogenically cooled filters are installed, but room for additional filters is included in the design. The next element is a rotating collimator-exchanger system that houses three Söller-slit collimations 6.4 cm wide and 17.8 cm tall, 10′, 25′, and 50′ full-width-at-half-maximum (FWHM) angular acceptance, along with an 9×18 cm^2^ *Open* position to provide an unobstructed beam with maximum intensity, or when using horizontal energy focusing.

## 3. Detailed Specifications

### 3.1 Monochromator Drum

The monochromator drum is 213 cm in diameter, with a 40.6 cm inner diameter to accommodate the (separate) monochromator systems. The angular range of the drum is from the straight-through position (for optical alignment purposes) to 115° scattering angle. The practical angular range will be from the smallest angle possible due to radiation considerations (~17°), to the largest angle possible given the geometrical constraints imposed by interference with other instruments and facilities. This will be ~75° on BT-7, and is expected to be the full 115° on the second instrument.

There are three additional components that have been designed for the monochromator drum. One is the stationary “pipe” that goes from the edge of the drum on the source side, to the center post. This is an essential piece of shielding, as it determines to a substantial extent the lowest achievable drum angle that can be used on the working instrument. It also contains a vertical magnetic field for polarized neutrons if a white-beam polarizer (such as ^3^He) for the incident beam becomes available. The maximum size of the neutron beam onto the monochromator is 20 cm high and 11 cm wide. The drum design itself can accommodate a beam considerably wider than 11 cm, and this allows substantial neutron and gamma shielding here.

**Fig 2:**
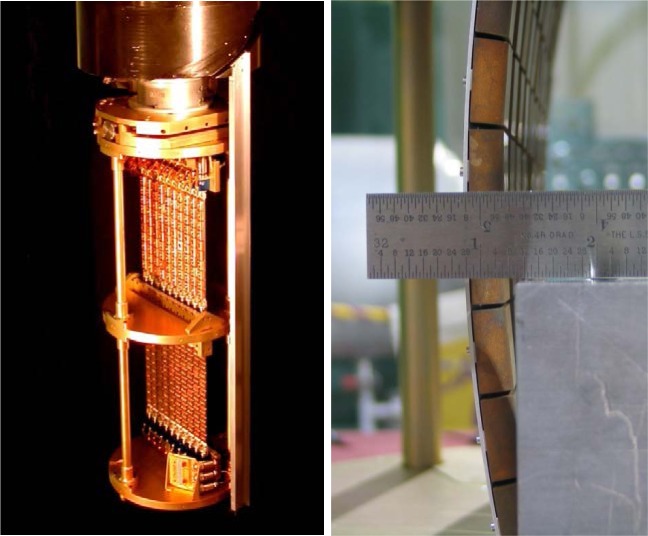
(Left) Double stack monochromator system. Each blade consists of 10 2×2 cm^2^ crystals, mounted on an aluminum plate that can be rotated to any angle to achieve the desired crystal alignment. Each top and bottom blade is coupled together and driven by a single motor. (Right) Vertical focusing is achieved by bending the Al plates (and then translating the array to maintain a centered monochromator), which is done under computer control. The Al plates are contoured so that the mechanical bending produces the correct cylindrical focusing condition [4].

The design of the double-focusing monochromator system was taken after the multiple blade design [1] that has been employed on the SPINS spectrometer [2], but with the addition of vertical focusing capability so that double focusing would be possible. An important aspect of the concept was to minimize the amount of material in the beam for the support structure. Initial efforts at development became delayed [3], and instead a separate cooperative research program was initiated to develop monochromator systems for both the new thermal and cold triple-axis instruments [4]. The double-stack system developed for the thermal instruments is shown in Fig. 2, with the PG(002) and cold-pressed Cu(220) monochromator crystals installed for the BT-7 instrument [5]. Each monochromator consists of 100 squares that are 2×2 cm^2^ each for a total height and width of 20 cm. They provide a continuous incident energy range on BT-7 from 5 meV (with PG) to 500 meV (with Cu). The monochromator systems are on an elevator and can be readily interchanged by computer control.

The beam optics for the monochromatic beam to the sample provides Söller-slit collimations of 10′, 25′, 50′, and 80′ FWHM, as well as open channels of various widths, depending on the sample size, for horizontal focusing. The beam width with collimation is 3.8 cm. The maximum sample size is designed to be ≈3 cm wide and ≈5 cm high. The shielding and masking system are tapered to achieve the desirable low instrumental background. Changes in collimation can be readily accomplished without the need to remove the sample and environmental system from the sample position. A guide field is also included for the neutrons exiting the drum for polarized neutron beam operation, if polarization capability at or before the monochromator becomes available. A low efficiency transmission detector located immediately outside the sample system monitors the flux of neutrons onto the sample.

**Fig. 3:**
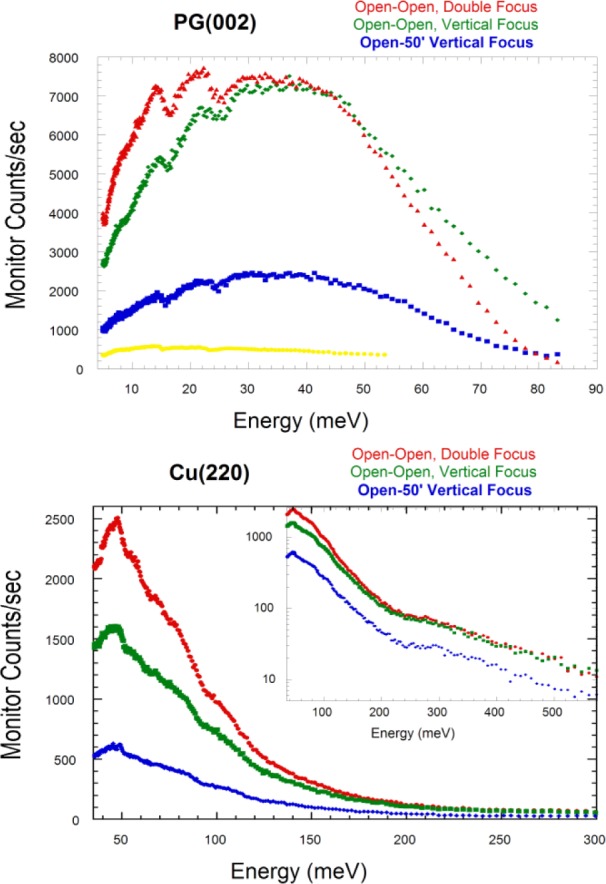
(Top) Monitor count rate versus energy for the PG(002) monochromator, using double focusing (red), compared with the monochromator flat horizontally and (variable) vertical focusing (green), and with vertical focusing and collimation of open-50 before and after the monochromator. Note that the primary gain in the intensity with double-focusing is the removal of the Söller collimation; then the advantage of using energy focusing is the improvement of the energy resolution. For comparison, the monitor rate for the old BT-2 TAS spectrometer (yellow) has been scaled by the measured flux at 14.7 meV (where the fixed vertical focus was optimized for BT-2); BT-9 had a similar flux. BT-7 provides more neutrons that also fully illuminate the sample over a wider range of incident energies, while the double focusing mode is seen to provide a dramatic gain in neutron intensity by relaxing the wave vector resolution of the instrument. (Bottom) Monitor rate using the Cu(220) monochromator system for various conditions as indicated. Neutron energies above 500 meV are available, but of course the flux drops off quickly at such high energies as shown in the inset.

For optimal vertical focusing of the monochromator onto the sample, the height of the beam *h_sam_* at the sample is related to the source height *h_source_* by [6]

(1) $$h_{sam}=\left(\frac{L_{M-S}}{L_{R-M}}\right)h_{source}$$

where *L_M-S_* is the distance from the monochromator to the sample and *L_R-M_* is the distance from the source to the monochromator [6]. Given a ≈5 cm high sample, the monochromator height was chosen to utilize the full height of the reactor beam available, while the width of the monochromator was chosen so that the sample width is fully illuminated over essentially the entire incident energy range of operation. Figure 3 shows the performance and energy range of the monochromator system for both the PG(002) and Cu(220) monochromators. Table 1 provides absolute flux numbers at a selection of energies and conditions.

### 3.2 Sample Stage

The sample table system is fixed to the monochromator drum in a cantilevered design (see Fig. 1), and consists of three concentric non-magnetic Huber axes for the sample rotation angle, horizontal field magnet axis, and scattering angle (2θ). The sample axis accommodates a Huber *x-y* tilt/translation goniometer for the sample and standard sample environment equipment. The sample axis is mounted on an elevator which has a vertical travel of +2.5 cm above the standard mounting-surface-to-beam distance of 15.25 cm, and 7.5 cm below. The goniometer is removed and the table lowered to its base to accommodate the 15 Tesla magnet which requires additional room in the vertical direction. The entire sample stage has a travel of 35 cm along the monochromatic beam direction (toward the monochromator). This can allow the experimenter to vary the maximum scattering angle from the sample, and to allow some flexibility when adding ancillary equipment. The default position is with the M-S distance a maximum to achieve a maximum scattering of 120° and incorporate maximum shielding along the M-S beam. A pair of *x-y* beam limiters inside the sample enclosure on the monochromator side move symmetrically about the nominal beam center both horizontally and vertically to reduce the size of the incident beam onto the sample. All motions are computer controlled. An additional manual masking system allows a mask to be placed immediately outside the sample environment system to further restrict the beam size.

With the exception of the openings for the incident beam and neutrons scattered toward the analyzer/detector system, the sample is surrounded by borated aluminum neutron absorbing shielding about 77 cm in diameter and 76 cm tall, to form a sample enclosure that both reduces experimental background and restricts access to the sample area when the beam is on. The beam stop immediately follows this sample enclosure to “catch” the incident neutron and gamma beams transmitted through the sample. The design is based on detailed calculations using the Monte Carlo N-Particle Transport Code (MCNP) to provide adequate shielding for health physics purposes under all operating conditions, and is basically a laminate of Pb and polyethylene separated by 0.125 cm thick plates of borated Al. For scattering angles below ≈17° the analyzer system moves behind the beam stop. To allow for low-angle operation of the analyzer system, the upper-front 15 cm thick portion of the beam stop is designed to lift up to allow the scattered beam through. In the area where the beam is transmitted there is 15 cm of single crystal Bi, located in the back of the beam stop at beam height, to attenuate gammas in the direct beam. The neutron shielding for the direct beam can be reduced in this case as the analyzer system itself provides the necessary additional shielding. Below about 10° scattering angle the scattered beam traverses the single crystal Bi, but detailed measurements show that there is no significant small angle scattering (beam broadening) from the Bi so that the measurement results are not compromised, other than a reduction in intensity of about 2 times.

**Fig. 4:**
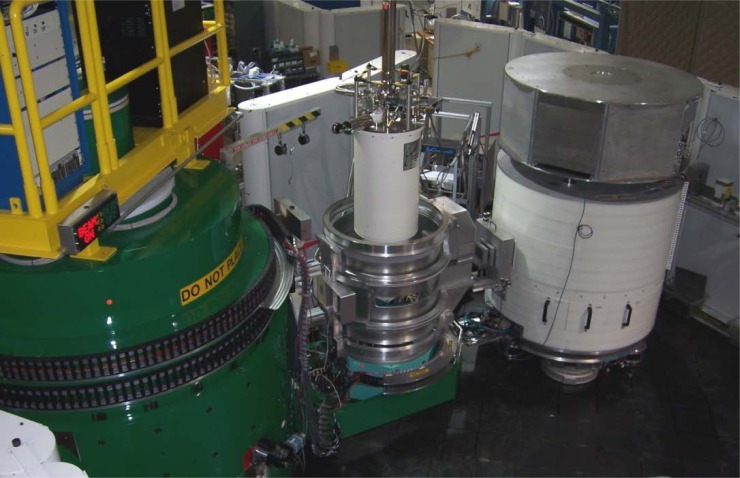
Photograph of the thermal triple axis instrument at BT-7 in operation. The electronics for the front portion of the instrument (through the sample position) resides on top of the (green) monochromator drum, while the sample environment system is the top-loading He^3^ system with a base temperature of 300 mK. The sample is surrounded by the sample enclosure to adsorb scattered neutrons and reduce ambient instrumental background. The analyzer system is below 17° so that the beam stop is in the up position. Two PG filters and an x-y slit system are on the x-rail in front of the analyzer system. The electronics for the analyzer system resides on top of the analyzer.

A photograph of the completed instrument is shown in Fig. 4, which shows the (green) monochromator drum, sample position with the top-loading He^3^ sample environment system in operation on the instrument, the beam stop system in the up position, and the analyzer.

### 3.3 Analyzer/Detector Systems

The design philosophy of the energy analyzer portion of the spectrometer is to allow several different styles of systems to be available and easily interchanged. Each system would be attached to the 2θ arm of the sample table through a pinned, hinge mechanism, which will allow modest vertical displacements while providing the lateral rigidity and alignment necessary to assure the proper angular precision. The essential feature is that the connection is designed in a “quick” coupling modular fashion so that the analyzer/detector systems can be readily accommodated and most importantly, quickly interchanged. To achieve this rapid interchangeability, the detector electronics and analyzer motor controllers must be integrated into each analyzer unit so that the only connections are power, a communications cable, and compressed air. The units move along the floor on an air-pad suspension to accommodate the weight and varied footprints of the sample-analyzer distance. The floor is a poured epoxy base covered by anodized aluminum tooling-plate tiles that are level to within ±0.75 mm over its entire area. The standard analyzer system, currently the only one available on BT-7, is a 13 vertical blade pyrolytic graphite analyzer system. A second proposed type of analyzer system would consist of a series of up to 30 individual and isolated analyzer/detector systems, where each analyzer/detector combination would be limited to a maximum of ≈75° for the detector (i.e. E_f_ = 5.0 meV minimum energy). This style of analyzer has now been adopted for the MACS double-focusing monochromator cold triple-axis instrument [8], but employs a double-crystal analyzer system for each detector in order to accommodate higher detector scattering angles needed for cold neutrons. Other analyzer options proposed include incorporating a velocity selector into the analyzer system, and developing a “conventional” analyzer/detector system where the analyzer crystals can be interchanged, double-focused, etc., and with a buried, well-shielded detector.

**Fig. 5:**
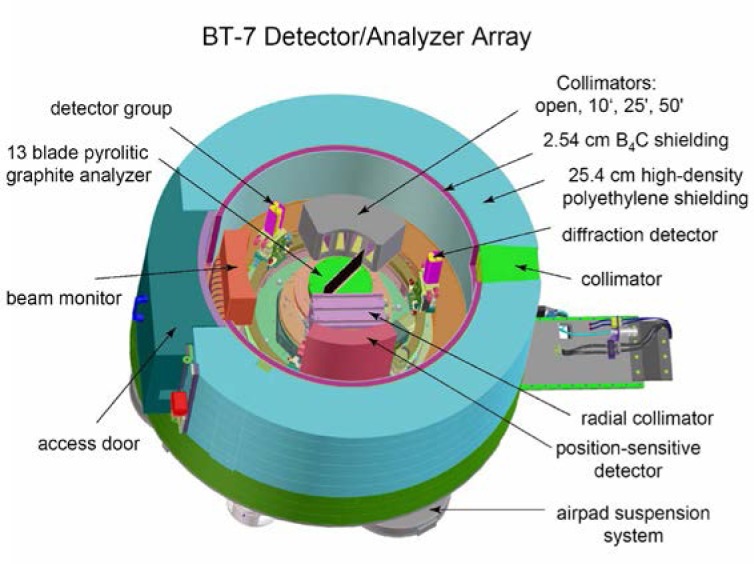
Schematic cutaway of the inside of the current analyzer system, showing the single detectors, PSD, radial and Söller collimators.

The analyzer system installed on BT-7 is shown schematically in Fig. 5. An x-rail is mounted externally to accommodate a variety of equipment between the sample and analyzer and is aligned toward the sample. Standard equipment includes computer controlled *x-y* beam-limiter slits, PG filters, a liquid-nitrogen cooled Be filter, and polyethylene shields for background reduction. Söller-slit collimations of 10′, 25′, 50′, and 80′, as well as open channels of various widths, are available and interchanged manually. In addition, radial collimators of 40′ and 80′ that accept a ≈5° angular range are available for diffraction and for horizontal focusing arrangements. The entire analyzer system can be translated by 300 cm under precision computer control to increase the sample-analyzer distance and accommodate additional components such as cold filters, ^3^He polarizers, and spin flippers.

Inside the analyzer assembly, the analyzer crystal system consists of 13 PG elements, each 2 cm wide and 15 cm high. The graphite element is mounted on a perfect Si single crystal 1 mm thick to minimize any phonon scattering, while the highly perfect extinction-limited crystal essentially eliminates Bragg scattering from the mount. The blades of the analyzer can be freely rotated by 360 degrees and individually positioned, while the entire unit can be rotated as a whole to achieve the desired focusing conditions as detailed below. A ‘single’ detector composed of three He^3^ detectors 2.5 cm in diameter and 15 cm high can be used as the signal detector, either with (up to) 13 blades of the analyzer focused on the detector, or with Söller-slit collimations of 25′, 50′, and 120′, or an open channel of 5×15 cm^2^. An Ordela 1348N linear position-sensitive detector is also contained in the system, along with an 80′ radial collimator matched to the analyzer system and PSD that focus to the sample position. The PSD detector has 48 individual wires covering an active area of 36° with a height of 16.5 cm. A separate diffraction detector identical to the ‘single’ detector is also provided, which can be moved in front of the analyzer if the energy-integrated signal is to be measured, for the measurement of Bragg peak intensities, or for alignment purposes. In addition, eleven 5×15 cm^2 3^He detectors are imbedded in the door behind the analyzer crystals to continuously monitor the neutron flux entering the analyzer system. These detectors are primarily used to check for intense scattering from the sample that might give rise to a spurious count rate in the signal detector, but they can also be used for measurements of the instantaneous (energy integrated) correlation function, for example, or with a radial collimator to determine a diffraction pattern over a limited angular range with coarse resolution (instead of the PSD).

The general design philosophy is to make the analyzer system as flexible and user friendly as possible while still meeting all the desired operating criteria. All the internal movements are computer controlled and all the various analyzer modes of data collection are obtained without any need for manual operations or user intervention inside the analyzer system, which is essential as the Si crystals holding the PG are very delicate. This type of analyzer system offers various ways to collect data depending on the scientific needs, and a similar capability is now available on the RITA-II spectrometer at the Paul Scherrer Institute [9–11].

## 4. Analyzer Modes of Operation

**Fig 6:**
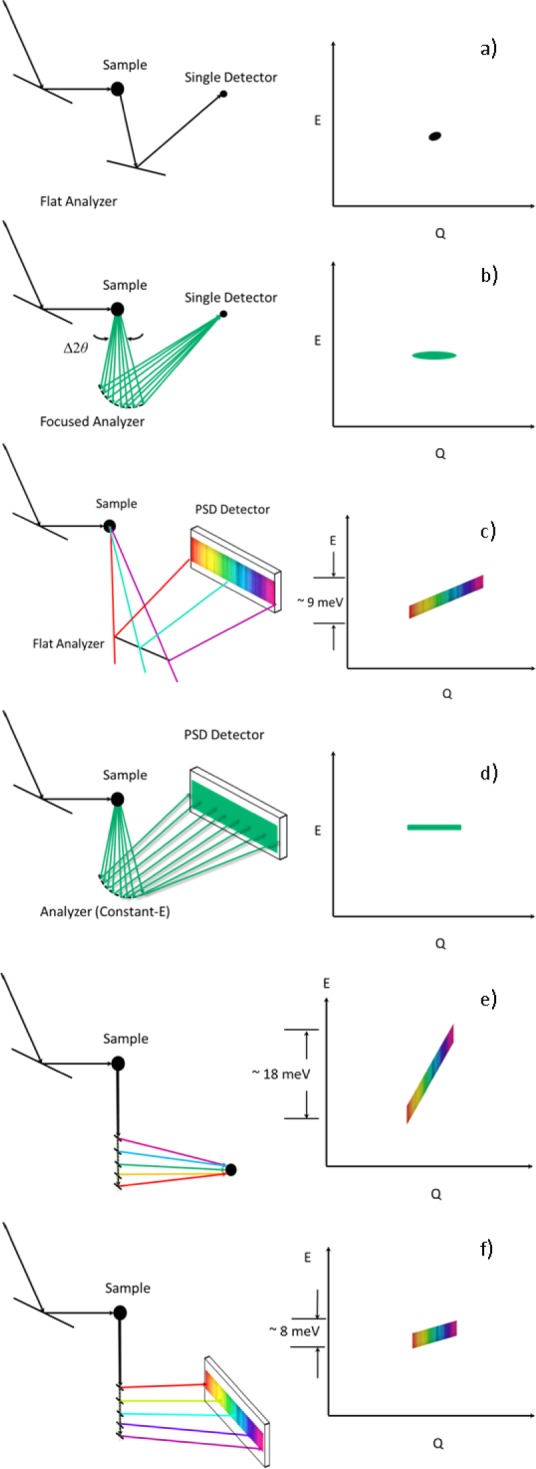
Different energy-analysis modes of the analyzer system, each representing a single setting of the spectrometer. (a) Conventional mode, measuring a single (**Q**,E) point with the single detector (or PSD). (b) Horizontal energy focusing mode, with either the single detector (or PSD). (c) flat analyzer array and PSD. (d) Constant-E_f_ mode with the PSD. (e) Constant-angle, integral E_f_ mode with the single detector (or PSD). (f) Constant-angle, variable E_f_ mode with the PSD.

The analyzer system has six basic modes of operation that are computer- controlled and can be selected and interchanged by the experimenter without requiring any mechanical reconfiguration or hands-on user intervention inside the analyzer housing. These measurement capabilities provided by the new analyzer system greatly increase the flexibility and ease of use of the spectrometer for the experimenter. The first two modes use the analyzer system without energy analysis, while the four standard additional modes use the analyzer and detector systems as indicated in Fig. 6. Additional configurations are also possible for specific measurement needs, two of which are also indicated in Fig. 6.

### 4.1 Diffraction Detector Mode

The simplest mode places a separate diffraction detector, composed of three 2.5 cm diameter single detectors 15 cm high and covering a width of 5 cm, in front of the crystal analyzer. This mode is used to align the scattering angle, calibrate the incident wavelength using standard powders such as Al_2_O_3_, Cu, or Si, and can be used to align single crystal samples. For data collection purposes, this mode can be used to make conventional Bragg peak intensity measurements of the sample. The advantage of this mode is that the detector is close to the sample and therefore provides a large vertical acceptance, while the monochromator vertical focusing and collimation can be chosen to assure that all the Bragg-scattered neutrons are counted.

### 4.2 Diffraction Mode with PSD

**Fig. 7:**
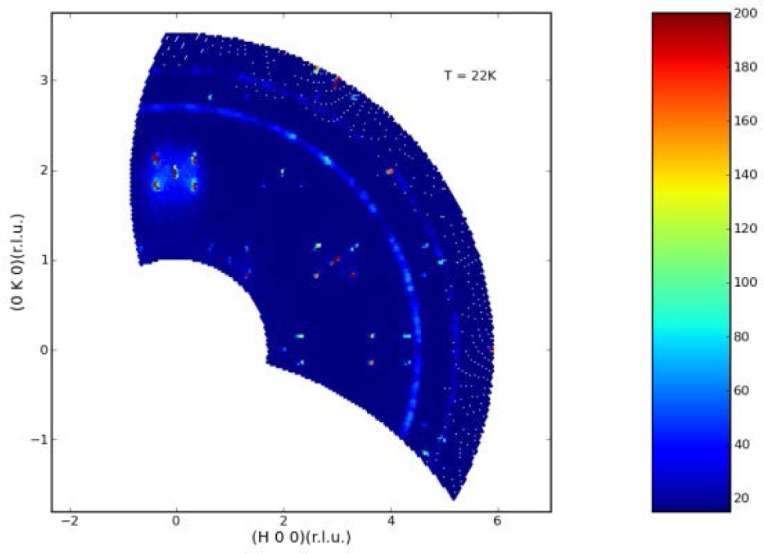
Observed scattering intensity for a single crystal of Co_3_TeO_6_ at 22 K, just below the antiferromagnetic phase transition of 26 K. Both commensurate and incommensurate magnetic peaks are observed for various temperature ranges from 2.5 to T_N_=26 K, and magnetic field ranges up to 14 Tesla. Note at this temperature there is also diffuse (energy integrated) inelastic scattering observed around the (0,2,0) peak [12].

This mode uses a radial collimator (40′ and 80′ FWHM are available) in the sample-to-analyzer (S-A) position and the position-sensitive detector (PSD) in the straight-through position to provide a simultaneous measurement of the scattering along an arc in reciprocal space for single crystal specimens. This will greatly accelerate making maps of diffuse scattering, for example, or may be used to monitor the intensity and position of a Bragg peak associated with a magnetic or structural phase transition. Software is available to convert the scattered intensity from (θ_s_, 2θ) to reciprocal space maps such as shown in Fig. 7 [12].

**Fig. 8:**
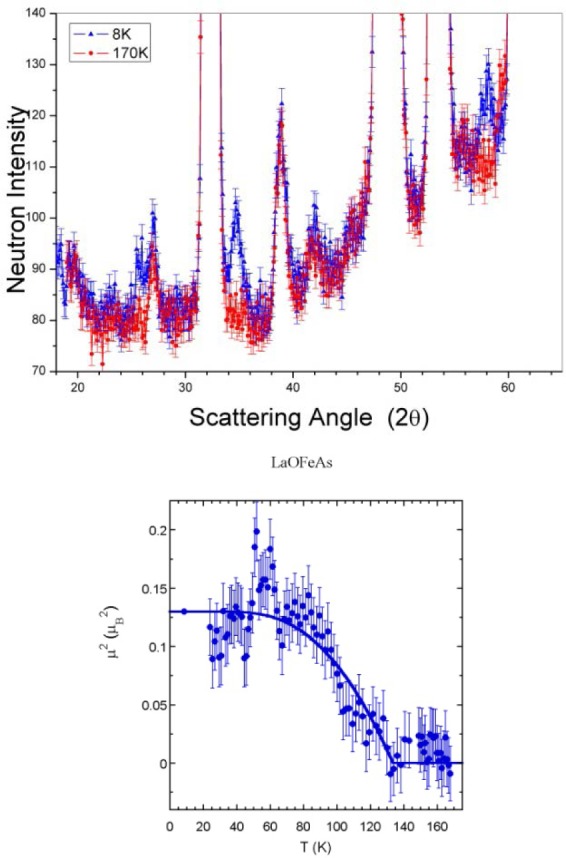
(top) Diffraction pattern for LaFeAsO indicating the development of weak magnetic peaks associated with the spin-density-wave ordering. (bottom) Temperature dependence of the intensity of the strongest magnetic peak, which is a direct measure of the square of the sublattice magnetization. These were the very first data obtained using this mode with the PSD [13]. Uncertainties where indicated throughout this article are statistical in origin and represent one standard deviation.

Alternatively, for a powder specimen a measurement of the diffraction pattern over a range of angles can be obtained simultaneously. This will greatly reduce the time to obtain a (coarse resolution) diffraction pattern, or can be used to quickly measure the integrated intensity of a single crystal Bragg peak associated with a magnetic or structural phase transition. An example is shown in Fig. 8 for LaFeAsO system, which exhibits magnetic order at 137 K, below the tetragonal-orthorhombic structural transition at 155 K [13].

### 4.3 Conventional Triple-Axis Mode

**Fig. 9:**
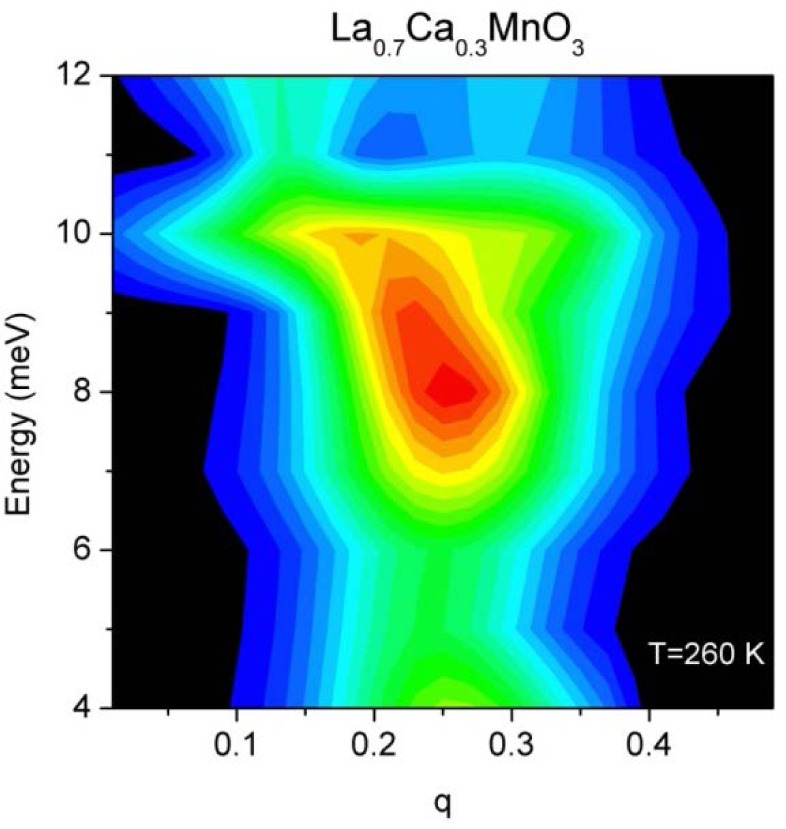
Map of the inelastic scattering in the paramagnetic state of the ferromagnetic (T_C_ = 250 K) single crystal of La_0.7_Ca_0.3_MnO_3_, showing the nature of the polaron correlations that emerge from the q=(¼, ¼, 0) positions [14].

This is the “standard” triple-axis configuration in which the analyzer is used in the flat configuration with Söller collimators before and after the analyzer. Typically then only about the five or fewer center blades of the analyzer reflect neutrons onto the detector, depending on the sample size and analyzer energy. One additional feature is that the door detectors can then be used to simultaneously determine the instantaneous correlation function. Figure 9 shows a map of the inelastic polaron correlations for the colossal magnetoresistive La_0.7_Ca_0.3_MnO_3_ material obtained at a mesh of points in this (**Q**, E) range [14].

### 4.4 Horizontal Energy Focusing Mode

**Fig. 10:**
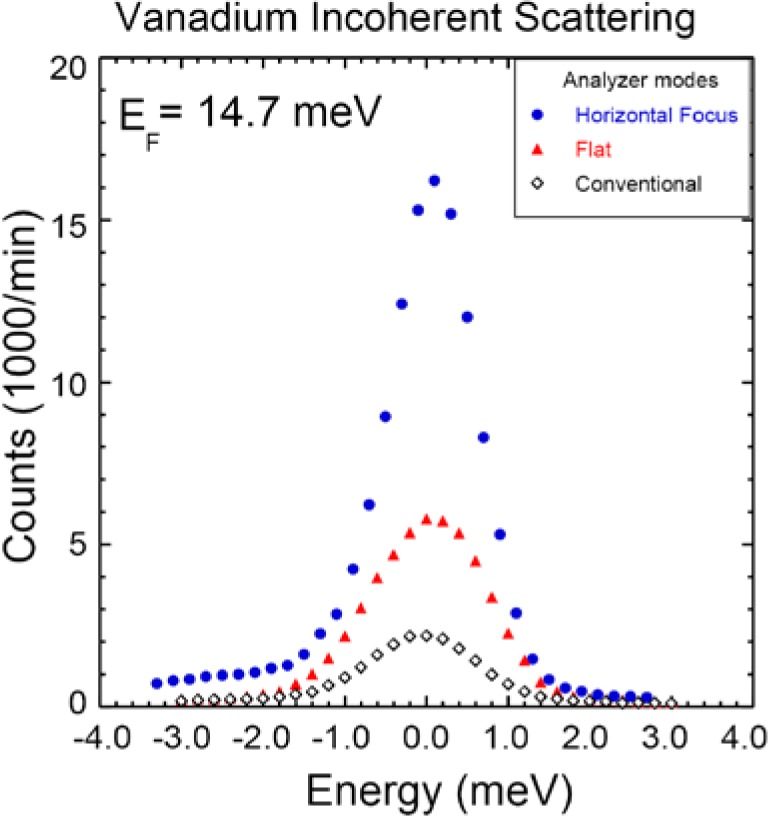
Energy scan through the incoherent scattering of a vanadium calibration sample, comparing the original (BT-2 and BT-4 style) analyzer temporarily used with the new instrument, with the new analyzer in flat mode, and with horizontal focusing. An improvement in performance of 2.4 is obtained over the old analyzer, which comes primarily from the increased analyzer/detector height. Relaxing the angular divergence by employing horizontal focusing mode provides an overall factor of 6.2 gain in signal.

**Fig. 11:**
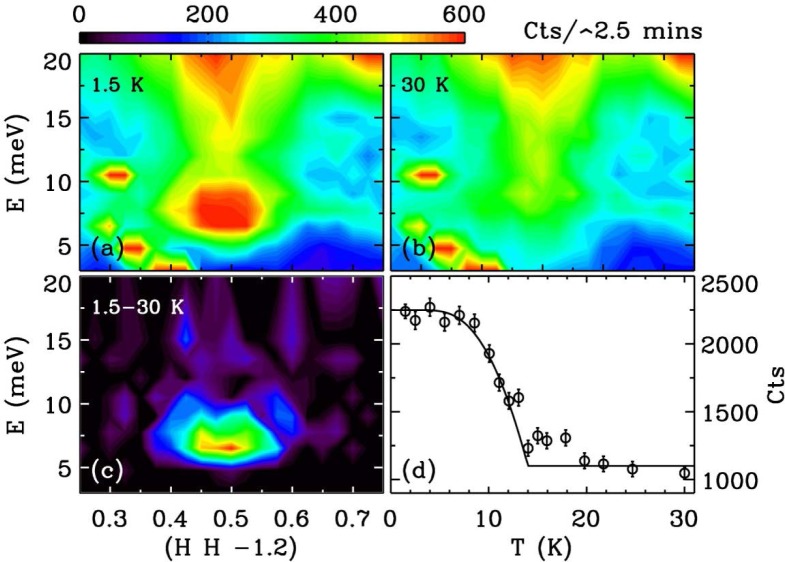
Magnetic inelastic scattering for the iron-based superconductor FeSe_0.4_Te_0.6_ taken with the analyzer in horizontal energy focusing mode. At 30 K (b) there is magnetic inelastic scattering, which acquires a strong resonance at 6.5 meV when superconductivity develops (a, c). The temperature dependence of the intensity of the resonance resembles the superconducting order parameter [15].

This mode employs a radial S-A collimator and the PG array in horizontal energy focusing mode with either the single detector or PSD depending on the measurement needs. For problems where the analyzer wave vector resolution can be relaxed this can triple the signal as shown in Fig. 10 using the elastic scattering from a vanadium standard. Figure 11 shows the dramatic ‘resonance’ signal obtained from a single crystal of an iron-based superconductor obtained using horizontal focusing [15]. This intensity of this magnetic resonance has an order-parameter-like dependence as the superconducting state develops.

**Fig. 12:**
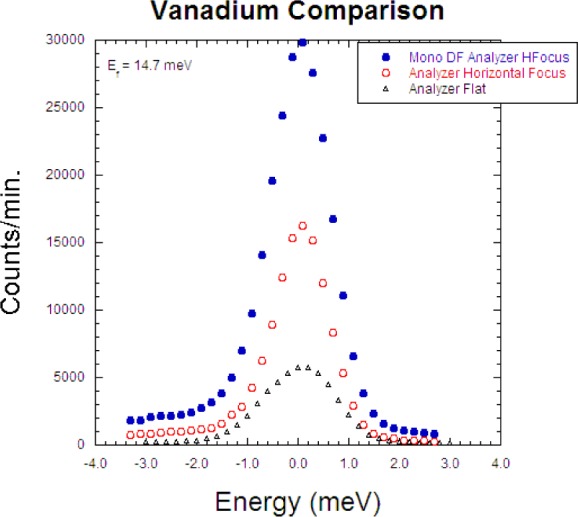
Demonstration of the intensity gain using horizontal focusing of the analyzer alone, and horizontal focusing of both monochromator and analyzer.

**Fig. 13:**
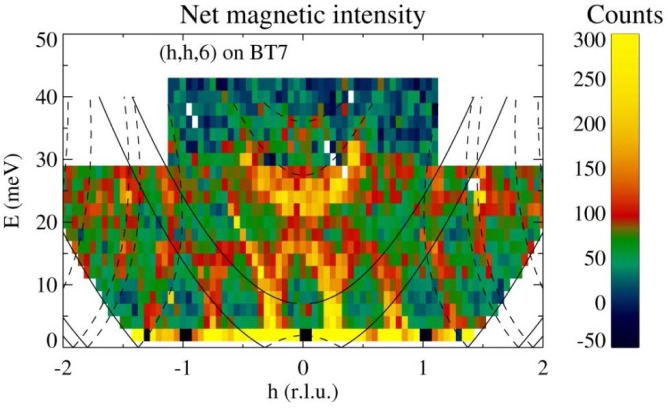
Magnetic scattering in the ground state for La_1.5_Sr_0.5_CoO_4_, using double focusing PG monochromator and horizontal focusing PG analyzer [16].

A further increase in signal can be obtained by employing double focusing with both the monochromator system as well as with horizontal focusing of the analyzer. The intensity comparison is shown in Fig. 12. An example using this option is shown in Fig. 13, where the inelastic magnetic scattering has been measured for La_1.5_Sr_0.5_CoO_4_ [16]. This material is an antiferromagnetic insulator, similar in structure to La_2_CuO_4_. At half doping, the lattice develops charge order below 825 K, where Co^2+^ and Co^3+^ ions form a checkerboard superstructure, while the spins are confined to the *a-b* plane and freeze below ~30K with short range order that is slightly incommensurate. At energies below ≈18 meV the magnetic scattering is dominated by a dispersive mode, which can be described either with a spin wave model or a spin liquid of interacting dimers. At higher energies, 20 meV < E < 32 meV, there is a broad continuum separated by a spectral gap. The short range nature of the magnetic correlations allows the resolution to be coarsened to employ both horizontal focusing modes without compromising the information obtained in the measurements.

### 4.5 Flat Analyzer with PSD (Q-E) Mode

**Fig. 14:**
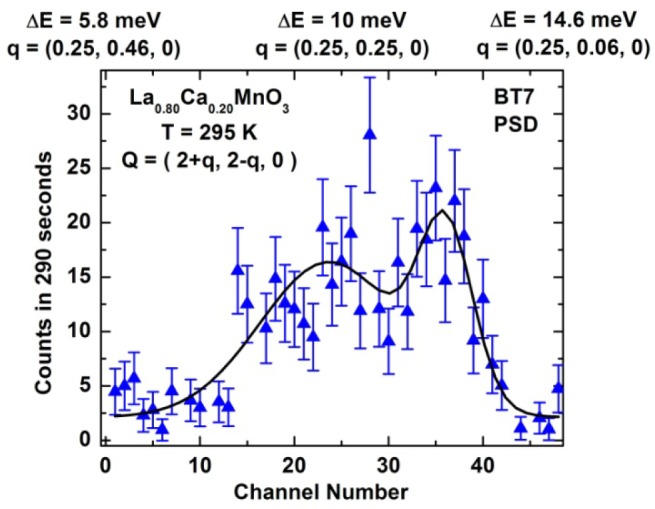
Inelastic scattering measured on La_0.7_Ca_0.3_MnO_3_ [14] for a single setting of the spectrometer. For this mode the Q and energy transfer are coupled together as shown on the top part of the x-axis.

This mode uses a radial S-A collimator, a flat PG array and the PSD to measure a range of (***Q***, *E*) simultaneously. Alternatively, one can use an open aperture between S-A, a radial collimator *after* the crystal analyzer, and the PSD. The signal-to noise is comparable for the two configurations, although at present the signal-to-noise is somewhat better with the first configuration due to better shielding along the S-A beam path. Fig. 14 shows the data collected for the same La_0.7_Ca_0.3_MnO_3_ sample as in Fig. 9 [14], at a single setting of the spectrometer centered at a wave vector of (¼,¼, 0) and energy transfer of 10 meV. This single setting of the spectrometer cuts through the entire scattering of interest to background on either side, and can thereby speed up the data collection rate by an order of magnitude. However, the energy and **Q** values are coupled together as indicated on the axes of the figure, so this is neither a constant-**Q** scan nor a constant-E scan.

### 4.6 Constant-E_f_ Mode

**Fig. 15:**
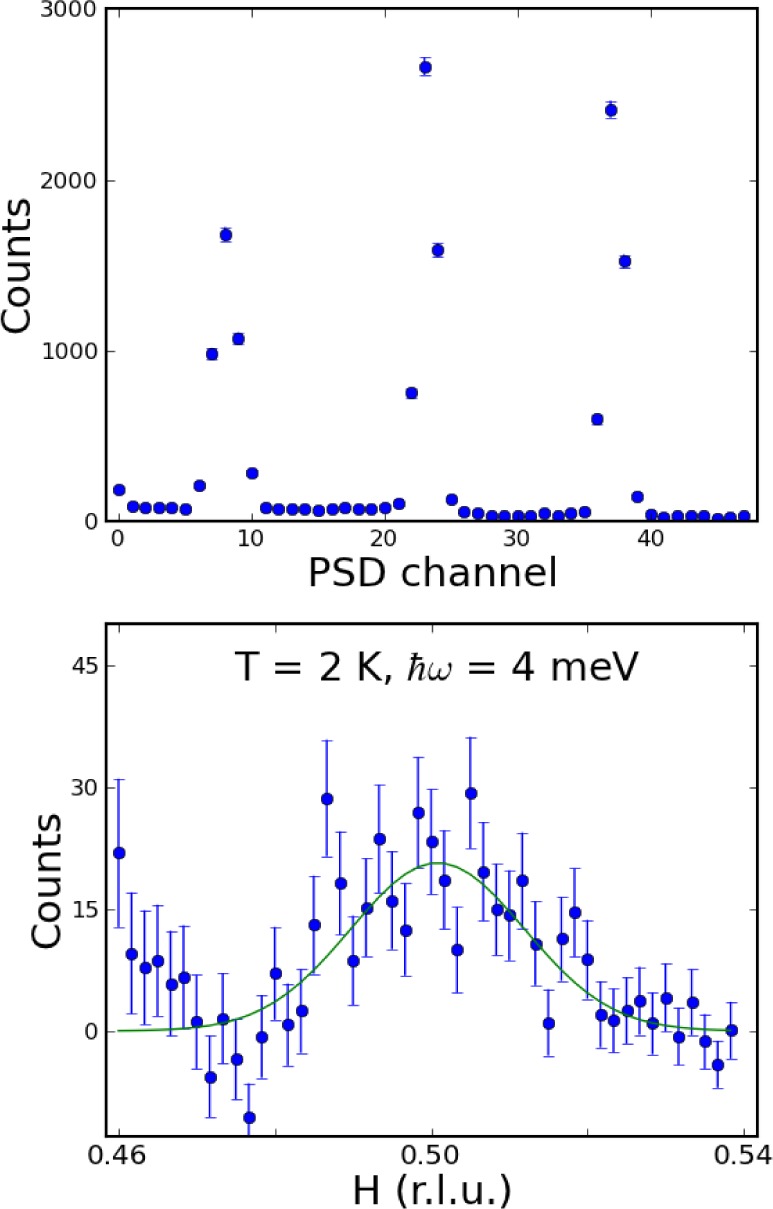
(top) Elastic incoherent scattering onto the PSD, with only three blades of the analyzer set for reflection at 14.7 meV, to demonstrate this mode of the analyzer. (bottom) Measured inelastic magnetic scattering for superconducting (Ba-K)Fe_2_As_2_ [17] at an energy transfer of 4 meV, for a single setting of the spectrometer. The approximate Q range for the constant-E mode is shown on the x axis, where a nice inelastic magnetic peak is observed centered at H=0.50. The upturn in scattering at smaller H is a background problem due to inadequate shielding.

For the usual mode of “horizontal energy focusing” demonstrated above in Sec. 4) the entire analyzer arrary is rotated so that the beam from each of the 13 analyzer blades is focused to the same point on the detector. Then you can use a single detector to collect all the neutrons, or you can use the PSD and see how well it focuses. In contrast to this customary horizontal energy focusing which integrates the scattering over wave vector, the idea here is to rotate the array away from this horizontal focus condition so that the scattering from each blade still scatters the same energy but falls on a different portion of the detector. In this way you get a constant-energy scan for each setting of the spectrometer, where each blade corresponds to a different **Q**. In terms of the scattering triangle, *k_f_* is the same length for each blade but varies over an angle of about 5° defined by the radial collimator. As a demonstration, Fig. 15 (top) shows the elastic scattering from an incoherent scatterer, where only three of the blades are aligned. Each peak is quite sharp, and corresponds to a different wave vector at the same final energy.

To demonstrate this measurement mode, we collected data on a single crystal of overdoped (Ba-K)Fe_2_As_2_ in the (H,H,L) scattering plane [17]. The data were taken by choosing the center point to be at the magnetic zone center position **Q**=(0.5,0.5,3), which is the wave vector position where the magnetic scattering is maximum, and then stepping the energy transfer over the range 1–20 meV. Background measurements were also taken for **Q**=(0.4,0.5,3) and **Q**=(0.6,0.5,3). Figure 15 (bottom) shows the signal at an energy transfer of 4 meV. These are the data for a single setting of the spectrometer, counted for 10 minutes, and nicely shows the expected ridge of magnetic scattering at H=0.5. This would take a several hours to measure using the “single point” method described in 3). The scale on the bottom shows the H coordinate (but note that L is also varying), with the center chosen to be (0.5,0.5,3). The upturn at small H for these demonstration data is a background problem due to inadquate masking of the detector, and should be easily remedied in the future.

**Fig. 16:**
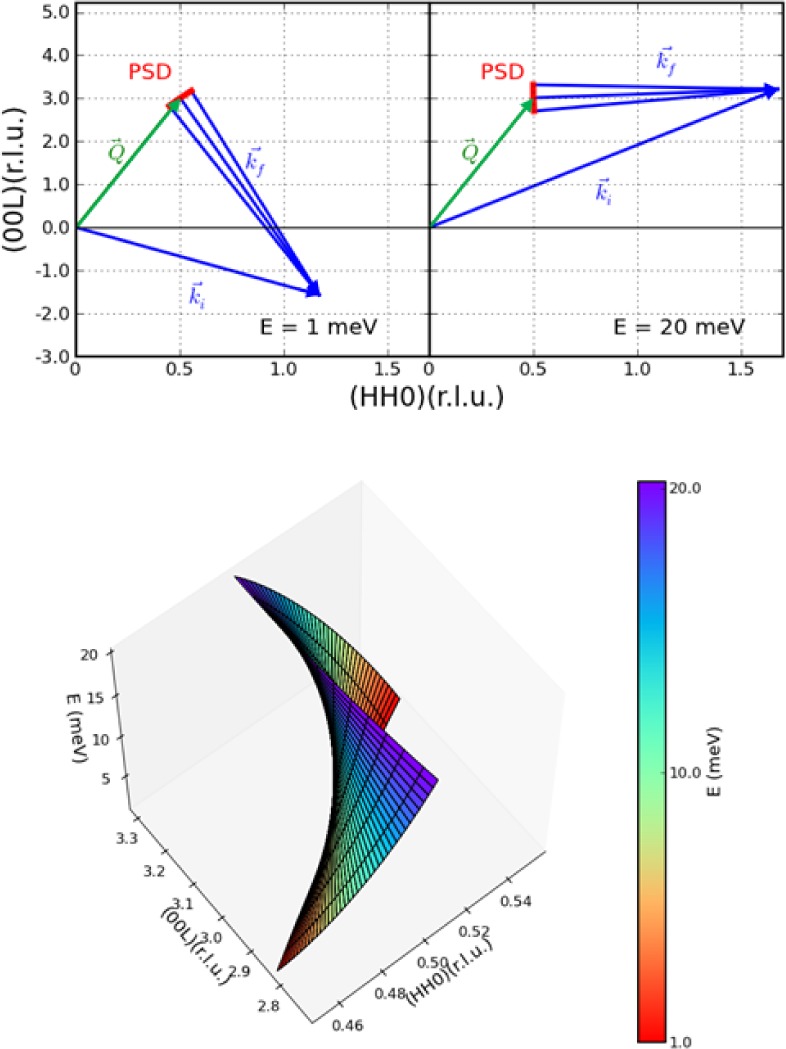
(top-left). The scattering diagram for this iron-superconductor crystal for an energy transfer of 1 meV, drawn to scale. The wave vector range sampled by the PSD at this setting of the spectrometer (red) is more along the [H,H,0] direction at low energies. At an energy transfer of 20 meV (top right) we see that the wave vector range sampled has rotated to along the [0,0,L] direction. (bottom) Isometric plot showing the relationship between the range and direction of the wave vector obtained at each setting of the spectrometer, as a function of the energy transfer indicated by the color bar, from 1 meV (red) to 20 meV (purple).

For the data shown in Fig. 15 (bottom) at an energy transfer of 4 meV, the wave vector range of the data is more along the (H,H) direction in this (H,H,L) scattering plane, as shown by the red line in Fig. 16 (top-left). However, as the energy transfer increases the **Q** range sampled by the PSD rotates, so that at an energy transfer of 20 meV the scan is essentially along L rather than along H as shown in Fig. 15 (top-right). The continuous range of wave vectors measured with this constant energy mode of operation is shown in Fig. 16 (bottom) as a function of energy.

Each of the modes described above have scripts to set up the modes on the spectrometer, and software in DAVE [18] to visualize and analyze the data. In addition to these capabilities, the flexibility of this blade design offers additional configurations that can be tailored to specific measurement needs. For example, Fig. 6(e,f) shows two configurations that use a Söller collimator in the sample-analyzer position, and the PG crystals oriented approximately along the direction of k_f_ but with each blade set to scatter a different energy. Fig. 6(e) shows a configuration where all the scattering is focused onto a single detector, while Fig. 6(f) uses the PSD to discriminate the (**Q**,E) values measured. This flexibility of the analyzer system can be employed to adjust the measurement conditions, such as the slope of the (**Q**,E) measurement, to take best advantage for a specific measurement need.

One final comment about these various modes. In comparing modes 4) and 6), for example, the counts detected using the PSD in mode 6) can be summed and are then equivalent to what is detected via mode 4). However, by using mode 4) the neutrons are focused on a small area on the PSD, or equivalently onto the single detector, which has the advantage that the active size of the detector is smaller, and presumably better shielded. Thus the signal-to-noise should be better using mode 4), but at the cost of losing the wave vector differentiation. The same argument can be made when comparing the configurations shown in Fig. 6(e) and (f).

## 5. Performance

The overall dimensions of the complete instrument as presently configured are: 1) source to monochromator distance, 488 cm; monochromator to sample distance, 206 cm; sample to analyzer distance, variable from 165 cm to 229 cm (default position); analyzer (center blade) to detector distance, 35 cm. The instrument can accommodate the full range of sample environment equipment to vary temperature, pressure, electric field, and magnetic fields. In particular, temperatures from 20 mK to 2000 K are available, and magnetic fields to 15 Tesla.

## 6. Polarized Beam Option

A polarized beam option has been developed for BT-7, utilizing ^3^He polarizers immediately before and after the sample [19]. This gives BT-7 full polarized beam capability for experiments where a guide field (only) is applied at the sample position to control the direction of polarization, and thereby the cross sections, with either monochromator and the PG analyzer. In particular, all the above configurations can be utilized with ^3^He polarizers before and after the sample, along with computer controlled polarization direction at the sample, to enable measurements of all eight of the conventional polarized neutron cross sections [20]. There are two spin rotators that can be mounted before and after the sample. Alternatively, the polarization of the ^3^He itself can be inverted to achieve the alternate spin state before or after the sample. An adjustable guide field at the sample position is under computer control to manipulate the polarization direction perpendicular or parallel to the scattering plane.

## 7. Operational Notes

The electronic systems for the new instrument are distributed among its components. Controls for the primary spectrometer (beam conditioning, sample table, monochromator drum, double focusing monochromator, scattering angle) reside atop the monochromator drum. All controls for the secondary spectrometer (analyzer motors, detector electronics, airpad controls), on the other hand, are housed in an enclosure on top of the analyzer itself. The only physical connections of the analyzer system to the rest of the instrument are a mechanical coupling, compressed air (for the air-pad system), electrical power, and computer communications. The distributed nature of the electronics and the simple linkage of the analyzer to the primary spectrometer are designed to both alleviate heavy cabling burdens and to facilitate interchangeability of the analyzer. In particular, if a different type of analyzer capability is required, such as a double focusing array with a detector buried in shielding, an analyzer with a different crystal choice, or a multi-crystal/multi-detector array, then the separate analyzer can be installed by floating it in on air pads, attaching it to the scattering angle arm, and simply connecting power, air, and communications. The future development of additional types of analyzers will add important measurement capabilities both for the thermal as well as for the cold triple-axis instruments. Current information about the instrument and many additional details of the operation are available on the instrument webpage [21].

## 8. Future Options

One of the drawbacks of the triple-axis spectrometer is that monochromator and analyzer crystals reflect higher order wavelengths, and these can not only contribute to background but also cause spurious peaks to occur in measurements. A PG filter in the incident beam can remove higher order wavelengths, but only at discrete values of the energy. One idea we investigated to try to alleviate this problem on the new instrument was to rotate the PG filter in the reactor beam to scatter the second-order wavelength, acting like a “pre-monochromator” to reduce the intensity of the higher order contamination. One could then vary the scattering angle of the “filter” to deplete the higher-order wavelength over a continuous range of energies. However, we found that the transmission through the PG of the primary wavelength was greatly reduced, making this impractical. We only mention this because for the configuration shown in Fig. 6(e), the first blade (closest to the sample) scatters a higher energy than subsequent blades, so that the transmission of the longer wavelengths through each blade of the analyzer may not be optimal. We note that for the configuration shown in Fig. 6(f) we have the opposite situation, with the each blade scattering a lower energy than subsequent blades, so there should be no significant transmission problem in this case.

The original design called for three separate low-background monochromator systems with three different d-spacings and corresponding energy ranges and resolutions; PG(002), Ge(311), and Cu(220). Shielding needs dictated that there is only room for two monochromators, and the initial choice for BT-7 was PG and Cu(220). One advantage of Ge(311), besides the different d-spacing, is the suppression of λ/2, and this will be the monochromator of choice on the second thermal TAS instrument. However, the recent availability of velocity selectors with a large beam acceptance and energies up to 60 meV may allow these to be incorporated into our thermal triple-axis instruments. In particular, there is sufficient room along the reactor beam of BT-7 to accommodate such a velocity selector, and this would be the ideal situation, providing a clean, truly monochromatic incident beam over a continuous energy range. This would represent a major advance in thermal triple-axis spectrometry.

Finally, we note that we plan to accommodate a four-circle goniometer on the new instruments. Coupled with the diffraction detector or PSD, this will greatly increase our ability to determine crystal and magnetic structures, as well as the nature of diffuse scattering and short range order. For inelastic scattering, this capability will enable measurements of excitations in different scattering planes without the need for remounting the crystal, greatly increasing the efficiency of data collection and the completeness of the data obtained.

## Figures and Tables

**Table 1:** Absolute flux values for the PG(002) and Cu(220) monochromators. *d*-spacing for Ge(311) has been included as the hot-pressed monochromator crystals [7] for that monochromator are under development.

	*d spacing (Å)*	*Energy (meV)*	*Collimation*	*PG filter*	*Flux (10^7^ n/cm^2^/s)*
PG(002)	3.3542	40	Open-50′	No	10
		40	Double Focus	No	18
		14.7	Open-50′	Yes	2.4
		13.7	Double Focus	Yes	4.6
Cu(220)	1.273	100	Open-50′	No	2.0
		50	Double Focus	No	6.1
Ge(311)	1.702				
